# Supplementary material for: Predictors of elevational biodiversity gradients change from single taxa to the multi-taxa community level
Source: Nat Commun. 2016 Dec 22;7:13736. doi: 10.1038/ncomms13736 (PMC5192166; doi:10.1038/ncomms13736)
Supplement: Supplementary Information — Supplementary Figures, Supplementary Tables, Supplementary Methods and Supplementary References [file ncomms13736-s1.pdf]

Supplementary Figures

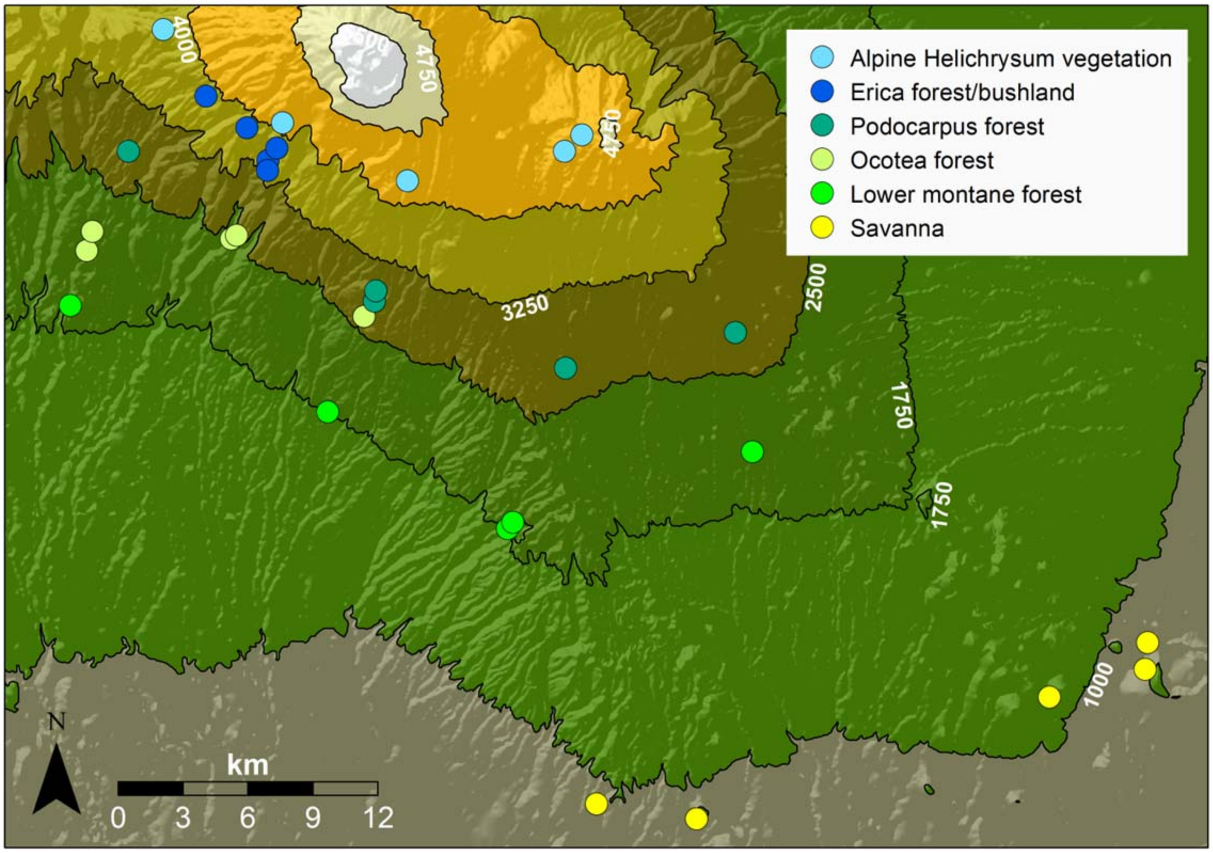

Supplementary Figure 1| Study area and distribution of study sites on Mt. Kilimanjaro.

Five replicate study sites were selected for each of the six major natural habitats on Mt. Kilimanjaro. The five study sites of each habitat type were distributed in a way to achieve a fine scale within-habitat elevational gradient.

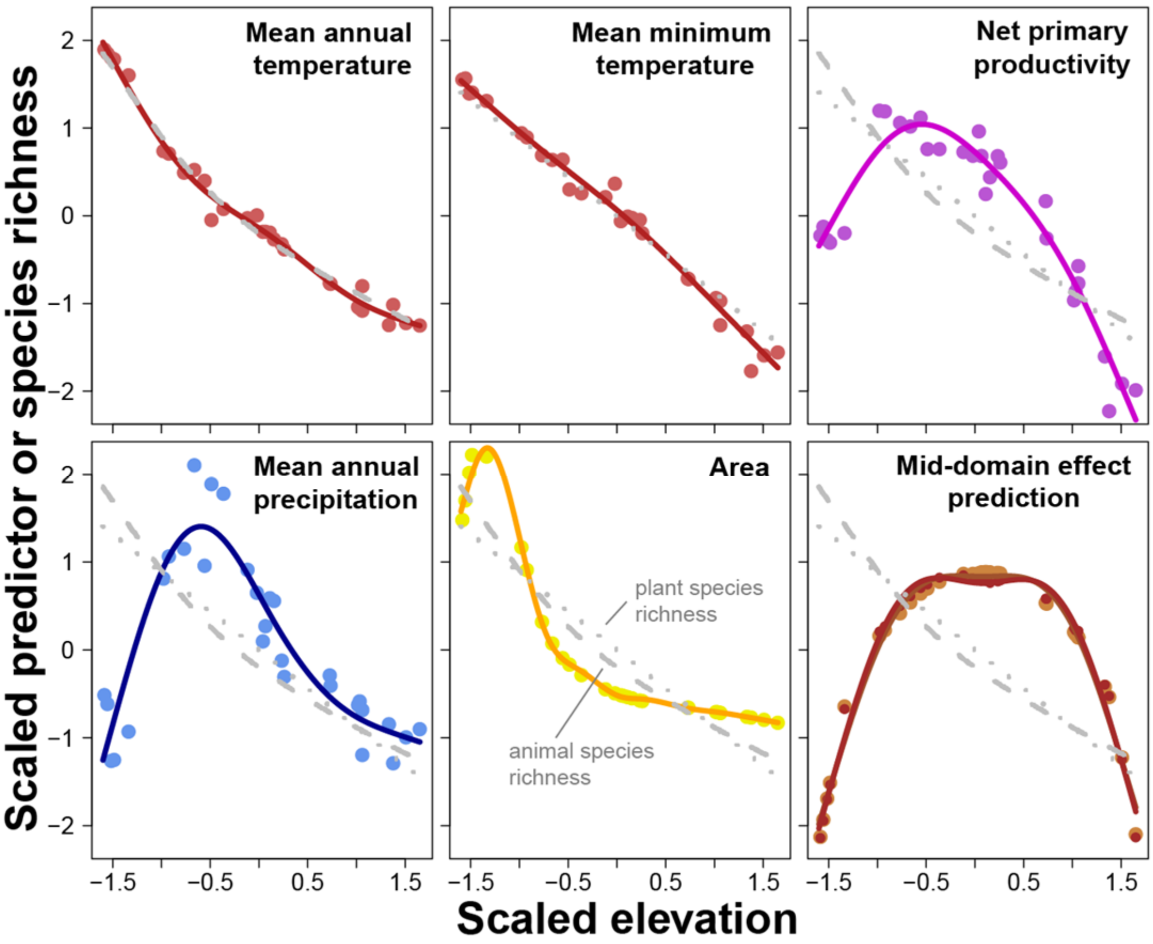

Supplementary Figure 2| Trends in predictor variables with elevation. Points indicate measures for individual study sites and lines are predictions of generalized additive models (generalized additive models, $N = 30$, $P < 0.05$). Trends in animal and plant elevational species richness are plotted as dashed lines. For reasons of comparability, all data were z-transformed.

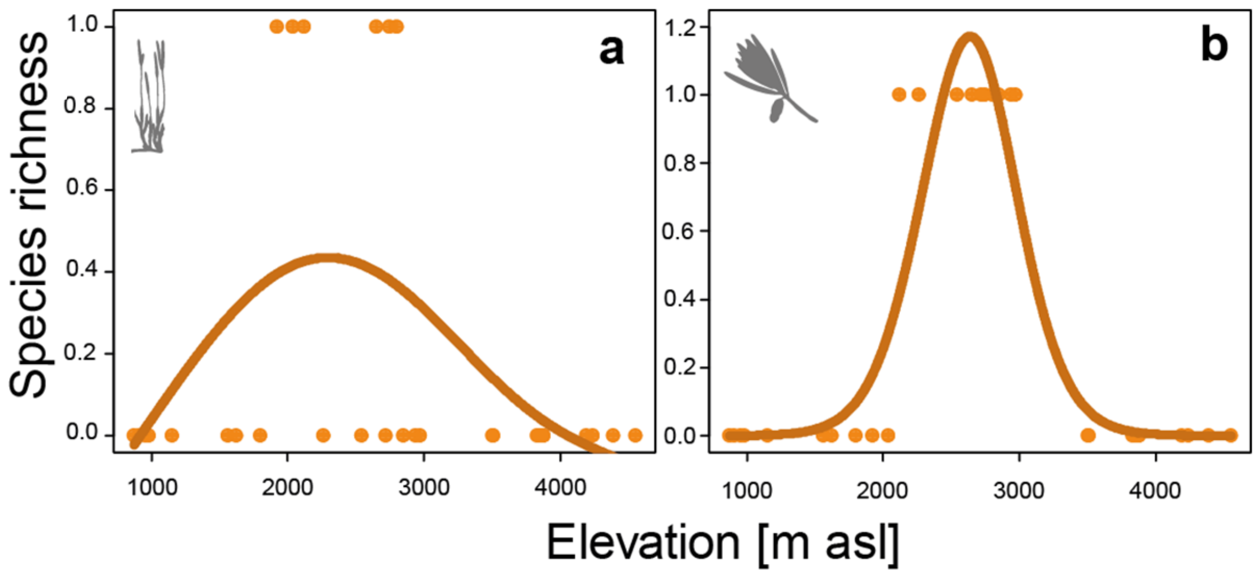

Supplementary Figure 3| Elevational species richness patterns of species-poor taxa. Shown are original species richness measures (dots) and predictions of generalized additive models (lines) for Lycopodiopsida (**a**) and conifers (**b**) (generalized additive models, $N = 30$, $P < 0.05$).

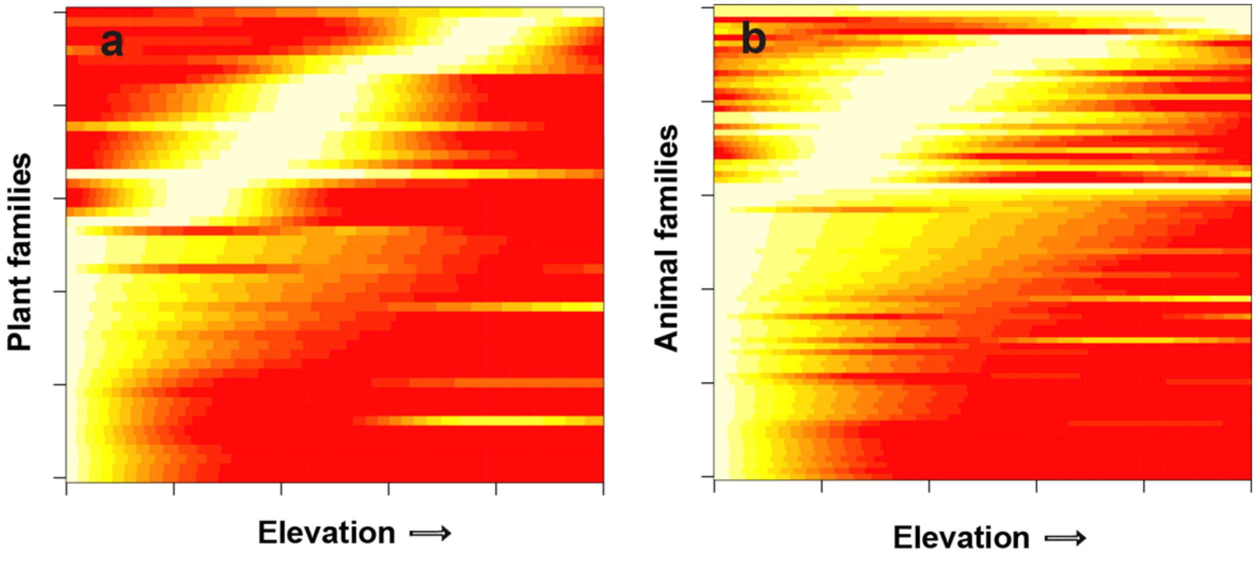

Supplementary Figure 4| Elevational species richness of single families. Shown is the distribution of species richness along the elevational gradients for individual plant (**a**) and animal families (**b**). Only families with more than four species were considered (i.e. 50 plant and 80 animal families). Each row shows the predicted species density of one family along the elevational gradient of ~800 to ~4600 m asl in heat colors (red = lowest species richness, bright yellow = highest species richness). Species richness of most families peaked at the lowest elevation. However, for families exhibiting hump-shaped distribution patterns, the respective elevations of highest species richness were variable and not centered in the mid-elevation of the gradient.

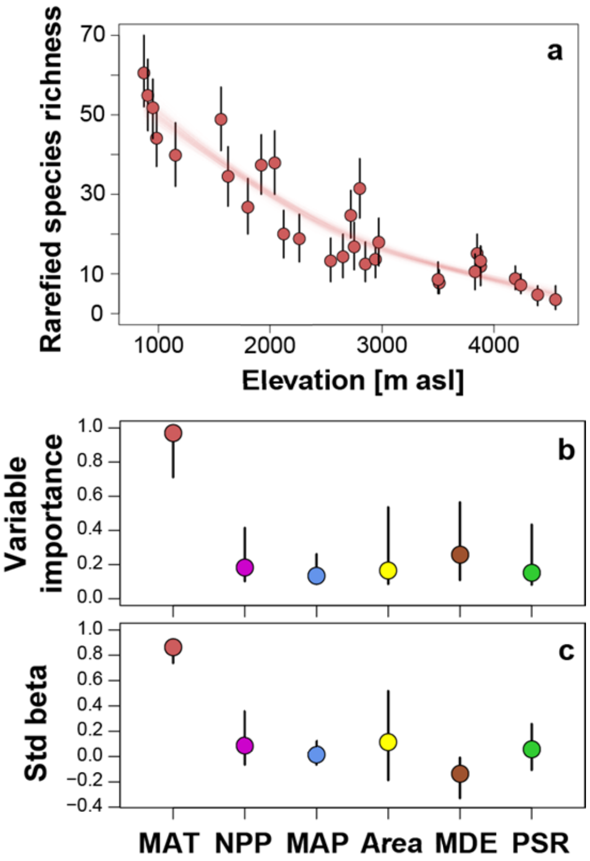

Supplementary Figure 5| Robustness of results. Differences in sampling intensity among animal taxa may have affected results found at the community-level. We therefore analyzed how standardizing the sampling intensity across taxa influenced patterns of elevational diversity (**a**) and the support of predictor variables (**b**, **c**). We repeatedly (N = 5000), randomly selected 83 individuals of each taxon (i.e. the number of individuals of the taxon with the lowest numbers of collected specimens, i.e. ‘other aculeate wasps’) and calculated for these rarefied data the mean and 95%CI of rarefied species richness for individual study sites (**a**, dots with s.e.m. bars) and model predictions of elevational diversity (**a**, lines) derived from generalized additive models. In Fig. 5a model predictions are shown for five hundred randomly selected data sets (lines). Additionally, we calculated for all 5000 rarefied data sets the support for individual predictor

variables using multi-model inference in the same way has done with the original data set. Figure 5b and 5c show the mean and 95%CI of variable importance and standardized beta values for each predictor variable. MAT = mean annual temperature, NPP = net primary productivity, MAP = mean annual precipitation, Area = area, MDE = mid-domain effect predictions, PSP = plant species richness.

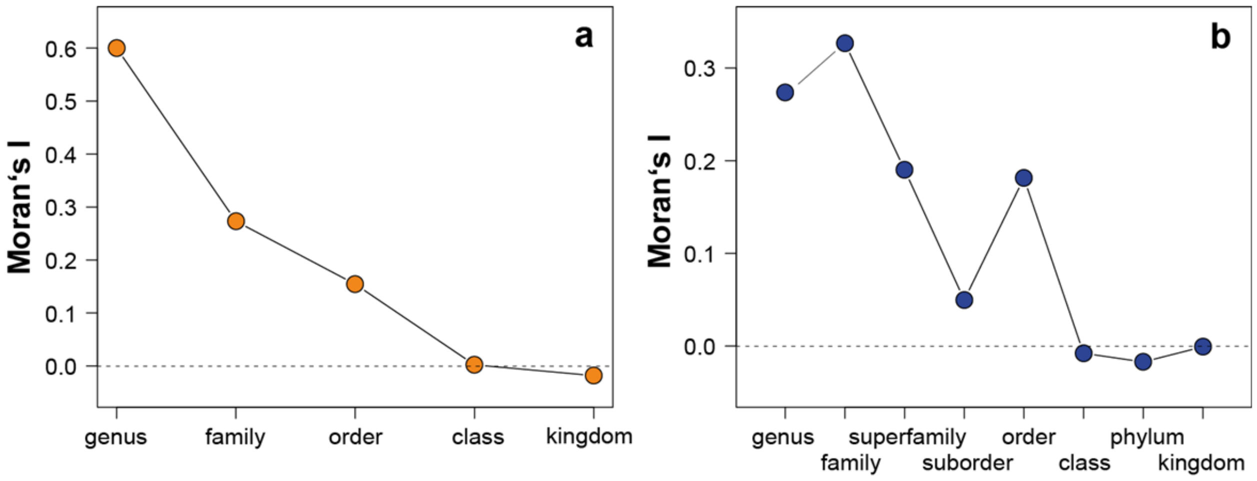

Supplementary Figure 6| Phylogenetic autocorrelation in elevational distributions. Correlograms show Moran's I values indicating levels of phylogenetic autocorrelation in the elevational distribution of plant (**a**) and animal species (**b**) at different taxonomic levels. The more strongly related species are the more similar is their mean elevational distribution. When we used the maximum and minimum of the range of species instead of the mean of the elevational distribution of species the figures looked very similar. In animals, calculation of Moran's I at the genus level was restricted to taxa which could be identified to genus level.

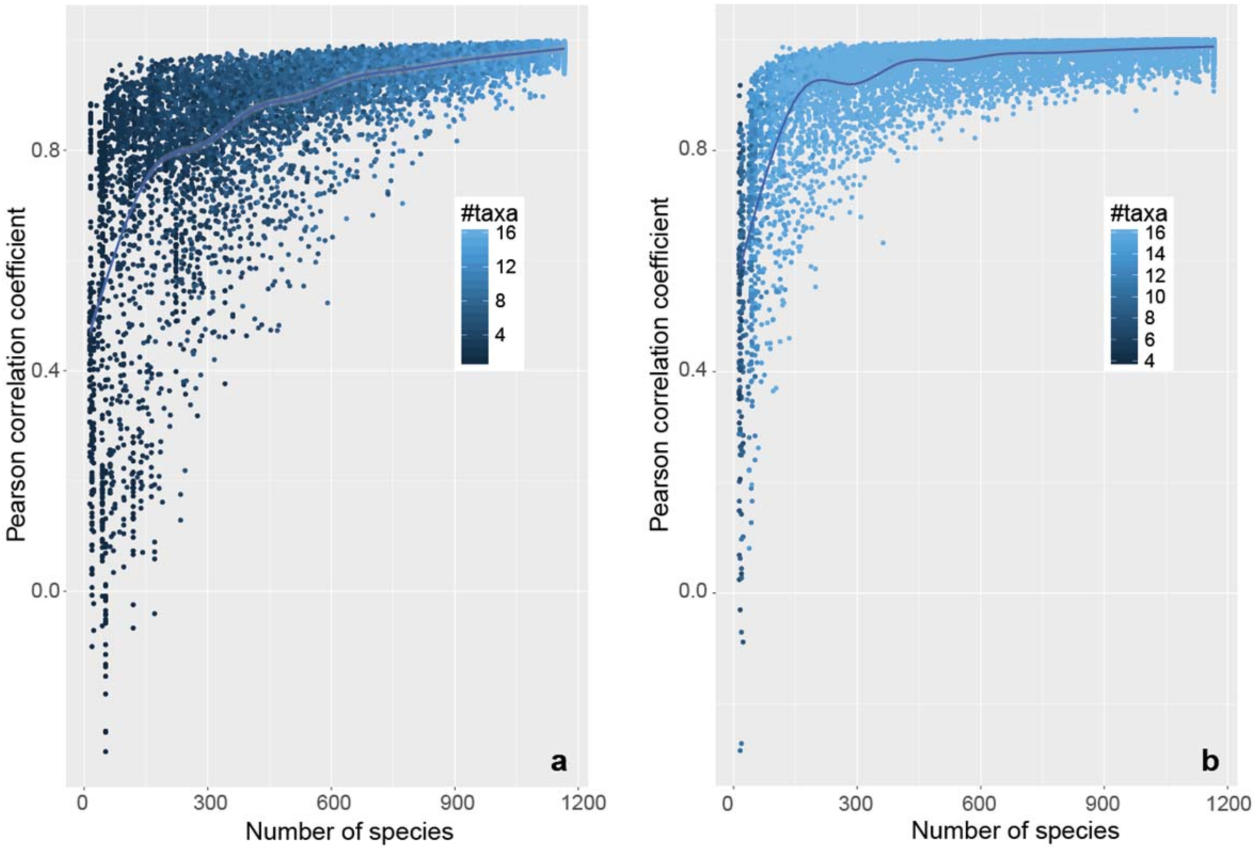

Supplementary Figure 7| Inferring community diversity by stratified random sampling. Shown are correlation coefficients for the correlation between the true community level richness and species richness estimates based on a stratified random sampling (**a**) or fully random sampling of species (**b**). For these analyses we assumed that the cumulative species richness of the 16 taxa sampled at 30 study sites along the Mt. Kilimanjaro elevational gradient represent the true richness of complete animal communities. In the stratified random sampling, one to 16 higher level taxonomic units (e.g. ants, bees, Collembola) were randomly selected first and their species numbers per study site were then assessed with a probability of 0.2, 0.4, 0.6, 0.8 or 1, simulating variation in sampling intensity among taxa (binomial probability function with the sampling probability randomly selected once for each taxonomic unit). The cumulative species

richness of all taxa was then correlated to the true community species richness. In the full random sampling approach, species were randomly selected from the whole species pool (without first selecting higher level taxonomic units). Species numbers of local assemblages (per study site) were then assessed with a probability of 0.2, 0.4, 0.6, 0.8 or 1 (binomial probability function). A random sample but also, slightly less efficiently, a stratified random sample of animal species gave a good representation of the community level diversity even when only a partition of their species or taxonomic units have been sampled. The sampling approach for animals at Mt. Kilimanjaro can be considered a stratified random sample of the community level richness, as the higher level taxonomic units and their respective sampling coverage were selected without any taxonomic bias, or hypothesis on elevational diversity in mind. Dots represent Pearson correlation coefficients for individual data sets. The lines show predictions of mean correlation coefficients derived from generalized additive models.

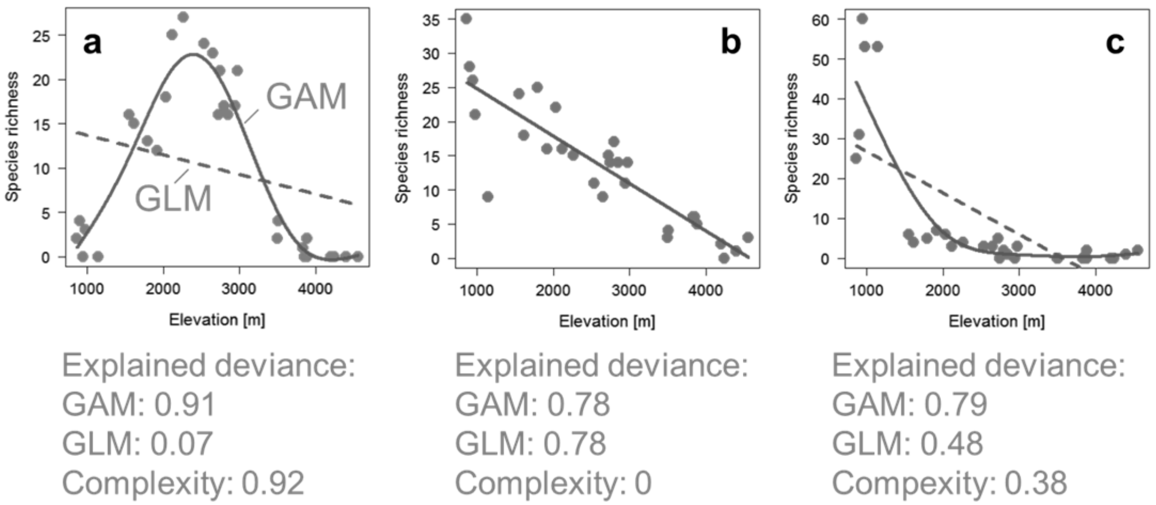

Supplementary Figure 8| Calculating complexity of elevational richness patterns. The three panels exemplify the calculation of the complexity of elevational species richness patterns (see Fig. 2). In ferns (**a**), which show a clear mid-elevation peak, the explained deviance of a generalized additive model (gam) is much higher than the explained deviance of a generalized linear model (glm), so that the complexity value is very high. In case of simple linear relationships, like in birds (**b**), gam and glm models show equal levels of explained deviance and the corresponding complexity value is therefore 0. Orthoptera (**c**), show intermediate levels of complexity in the elevational species richness pattern as the explained deviance of the gam model is only moderately higher than the explained deviance of a glm model. Complexity values for other taxonomic groups shown in Fig. 1 are: ferns = 0.92, magnoliids = 0.87, monocots = 0.61, other eudicots = 0.78, asterids = 0, rosids = 0.16, gastropods = 0.87, Collembola = 0.62, Orthoptera = 0.41, bees = 0.40, parasitoid wasps = 0.41, non-apid aculeate hymenoptera = 0.42, bees = 0.33, ground-dwelling beetles = 0.90, moths = 0.32, hoverflies = 0.75, amphibians = 0.11, birds = 0, aerial insectivorous bats = 0.

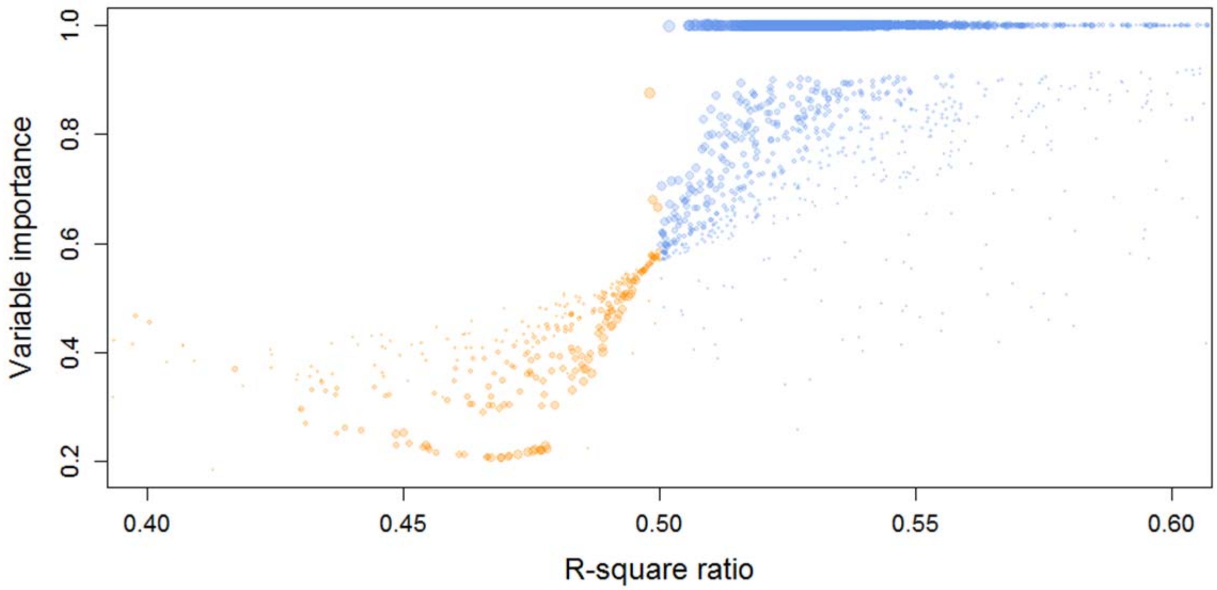

Supplementary Figure 9| Properties of multi-model inference under high levels of correlation among explanatory variables. To demonstrate the sensitivity of variable importance values towards increasing levels of correlation among explanatory variables we created 2000 artificial variables of species richness, in which species richness was explicitly determined by mean annual temperature (MAT) [species richness ~ 0.3 * MAT (original field data)] with an error distributed normally with a zero mean and a standard deviation of 0.1 to 0.5. The resulting response variables varied in their degree of correlation to MAT (R^2: 0 – 0.96) and due to co-correlation with MAT (r = 0.93) also with area (R^2: 0 – 0.93, for area also original variables were used). We modelled these artificial species richness variables as a function of MAT and area, using the original explanatory variables, in a simple additive linear model and used multi-model inference based on information theory to calculate levels of variable importance in the same way as done for the original data. We calculated simple linear regressions of the response variables on either MAT or area and calculated R^2 values from these

regression models. We expect the methodology to be adequate if variable importance values decline when levels of variance explained by MAT and area become similar. The dot plot shows the variable importance of MAT in relationship to the relative R^2 values of MAT and area [R^2-ratio = R^2_T / (R^2_T + R^2_{area})]. The color of dots indicates whether the variable importance of MAT was higher than the variable importance of area (higher = blue, lower = orange).When the explanatory power of MAT is considerably higher than the explanatory power of area (R^2-ratio > 0.5) the variable importance of MAT is high (e.g. > 0.8). In case of area having the same explanatory power as MAT (i.e. R^2-ratio = 0.5), variable importance values of MAT decline to levels of 0.5 indicating high levels of uncertainty to identify the 'correct' predictor of species richness. Variable importance of MAT declines to levels below 0.5 when R^2_T becomes smaller than R^2_{area}.

Supplementary Tables

Supplementary Table 1| Sample coverage of taxa. Shown are estimates of sample coverage of all animal species calculated with the r-package iNEXT[1]. Sample coverage is a measure of sample completeness, giving the proportion of the total number of individuals in a community that belong to the species represented in the sample[1]. Subtracting the sample coverage from unity gives the probability that the next individual collected belongs to a species not previously collected in the sample. 'NA's indicate study sites where not a single specimen of a taxon was found.

	Taxonomic group														
Study site	Other aculeate Hymenop.	Ground-dwel. ants	Aerial insectiv. bats	Bees	Ground-dwelling beetles	Birds	True bugs	Collembola	Dung beetles	Gastropods	Millipedes	Moths	Parasitoid wasps	Spiders	Syrphid flies
fer0	1.00	NA	NA	1.00	0.67	1.00	1.00	1.00	NA	1.00	NA	1.00	0.77	0.63	1.00
fer1	1.00	NA	1.00	1.00	0.62	1.00	0.58	1.00	NA	1.00	NA	0.49	0.91	0.76	1.00
fer2	1.00	NA	1.00	1.00	NA	1.00	NA	NA	NA	1.00	NA	NA	0.86	NA	1.00
fer3	NA	NA	NA	1.00	0.79	0.96	1.00	1.00	NA	1.00	NA	NA	0.74	0.65	1.00
fer4	NA	NA	0.93	1.00	0.56	0.85	1.00	1.00	NA	1.00	NA	0.67	0.83	0.44	NA
flm1	0.33	0.93	0.92	0.89	1.00	0.98	0.58	0.98	0.97	1.00	0.67	0.43	0.64	0.73	0.93
flm2	NA	0.67	0.86	0.76	0.84	0.95	0.67	0.98	1.00	1.00	NA	0.03	0.36	0.86	0.90
flm3	0.33	1.00	0.95	1.00	0.88	0.95	0.63	0.99	0.63	1.00	0.84	0.60	0.48	0.70	0.91
flm4	NA	0.83	0.97	0.58	0.80	0.97	1.00	0.97	1.00	0.98	1.00	0.51	0.45	1.00	0.88
flm6	1.00	0.63	0.84	0.63	0.86	0.98	0.76	0.88	1.00	1.00	1.00	0.13	0.66	0.73	0.67
foc1	NA	NA	0.83	NA	0.97	0.98	NA	0.99	NA	0.98	1.00	0.83	0.40	0.97	0.88
foc2	NA	NA	0.67	0.49	0.95	0.98	0.67	0.99	0.67	0.98	NA	1.00	0.41	0.66	0.76
foc3	NA	NA	0.89	0.92	0.93	0.97	1.00	1.00	NA	0.98	NA	0.67	0.07	1.00	1.00
foc4	NA	NA	0.92	1.00	0.84	1.00	NA	0.98	NA	0.98	NA	0.67	0.05	1.00	0.67
foc5	NA	NA	0.94	1.00	0.78	0.98	1.00	0.98	NA	0.98	NA	0.67	0.66	0.78	1.00
fpo1	NA	NA	0.18	NA	0.94	0.98	NA	1.00	NA	0.96	NA	0.67	0.63	0.79	0.76
fpo2	NA	NA	0.93	NA	0.94	1.00	NA	0.99	NA	0.97	NA	0.82	0.39	0.68	1.00
fpo3	NA	NA	1.00	NA	0.85	0.96	1.00	0.98	NA	1.00	NA	0.63	0.49	0.70	1.00
fpo4	1.00	NA	0.87	0.96	0.96	1.00	0.76	0.98	NA	1.00	NA	0.65	0.29	0.83	1.00
fpo5	1.00	NA	1.00	0.92	0.86	0.98	1.00	1.00	NA	0.99	0.63	0.56	0.68	0.95	0.78
hel1	1.00	NA	1.00	1.00	0.58	1.00	1.00	1.00	NA	1.00	NA	0.67	0.83	0.90	1.00
hel2	NA	NA	NA	1.00	0.49	1.00	1.00	0.93	NA	1.00	NA	NA	1.00	0.81	NA
hel3	0.83	NA	NA	1.00	1.00	NA	NA	1.00	NA	1.00	NA	NA	1.00	0.77	1.00
hel4	1.00	NA	NA	1.00	0.63	1.00	NA	1.00	NA	NA	NA	NA	1.00	0.63	NA
hel5	NA	NA	NA	1.00	0.90	1.00	NA	1.00	NA	NA	NA	NA	0.67	0.78	NA
sav1	0.40	0.81	0.85	0.92	0.58	0.95	0.66	0.96	0.95	1.00	1.00	0.90	0.02	0.33	1.00
sav2	0.28	0.86	0.97	0.93	0.62	0.95	0.73	0.83	0.93	1.00	1.00	0.78	0.40	0.11	1.00
sav3	0.05	0.88	0.73	0.84	1.00	0.98	0.39	1.00	0.87	1.00	1.00	0.68	0.32	0.67	1.00
sav4	0.18	0.60	0.94	0.95	NA	0.93	0.79	0.98	0.98	0.99	NA	0.11	0.07	0.33	1.00
sav5	0.04	0.79	0.98	0.91	0.51	0.99	0.74	0.99	0.96	1.00	NA	0.47	0.21	0.76	1.00

Supplementary Table 2| Test of the influence of sampling biases on elevational patterns of species richness. Observed and Chao1-estimated species richness values were modelled as a function of elevation using generalized additive models with a basis dimension of five. Based on these models, predictions of species richness for each site were calculated. Pearson's r give the coefficients of correlation of species richness predictions based on observed and the chao1-estimated species richness. Values near one indicate highly similar patterns of elevational species richness. Data in the column ESR pattern indicates the type of elevational richness pattern based on Chao1-estimated species richness. Under 'Notes' we described all changes in elevational species richness patterns based on Chao1-based species richness estimates in comparison to those presented in Fig. 1 of the main manuscript.

Taxon	Pearson's r	ESR pattern	Notes
Gastropods	0.99	Unimodal	Same pattern
Millipedes	0.99	Unimodal	Same pattern
Spiders	0.66	Bimodal	Similar pattern but higher richness in savannah and lower in upper montane forests
Collembola	1.00	Unimodal	Same pattern
True bugs	1.00	Exponential decline	Same pattern
Parasitoid wasps	0.98	Unimodal	Similar pattern; estimated richness in savannah slightly higher, at elevations above 3000 m asl slightly lower
Ground-dwelling ants	1.00	Exponential decline	Same pattern
Bees	0.98	Exponential decline	Similar pattern; slight increase in forests of 1500-2300 m asl and slight decrease in savannah
Other aculeate Hymeoptera	1.00	Exponential decline	Same pattern
Ground-dwelling beetles	0.91	Unimodal	Similar pattern; Slightly higher species richness in lowlands, lowered richness in mid-elevations and slight increase in highest elevations
Dung beetles	1.00	Exponential decline	Same pattern
Moths	0.95	Quasi-linear decline	Similar but less exponential pattern, more linear
Hoverflies	0.98	Unimodal	Similar pattern but slight increase in forests of 1500-2000 m and slight decrease in 2500-3000 m asl
Birds	1.00	Linear decline	Same pattern
Aerial insectivorous bats	1.00	Linear decline	Same pattern

Supplementary Table 3| Synthesis models explaining richness patterns of species-poor plant taxa. Shown are results of multi-model averaging models for Lycopodiopsida and conifers. .

			Conditional standardized estimates$^{\beta}$				
Taxon	#species*	#models†	MMT	NPP	MAP	Area	MDE
Lycopodiopsida	4	7	0.55	-0.10	0.54	-0.55	0.03
Conifers	1	8	1.19	-0.30	-0.26	-1.35	0.47

Shown are standardized parameter estimates of predictor variables derived from weighted averaging of parameter estimates over best-fit models. Colors indicate significant ($P < 0.05$) positive (blue) or negative (red) effects in multi-model averaging analyses.

*total number of detected species/morphospecies for each taxon

†number of best-fit models (ΔAIC<4) used for inference on parameter estimates and variable importance.

$^{\beta}$Standardized parameter estimates (standardized beta) over all best-fit models including the respective predictor variable.

Predictor variables: MMT = Mean minimum temperature, NPP = net primary productivity, MAP = mean annual precipitation, MDE = mid-domain effect prediction.

Supplementary Methods

Sampling protocols for studied taxa: Vascular plants [Tracheophyta; data owner: A.H.]: Plant species richness was assessed on one 20 x 50 m subplot per study site using the method of Braun-Blanquet[2]. Plant formations without seasonal variation in the presence of species (e.g. forests, alpine vegetation) were surveyed only once. Vegetation types with high seasonal variation and high proportions of annuals (savannah) were surveyed several times.

Ground-dwelling ants [Formicidae; data owner: M.K.P.]: Ant species richness was assessed using a diverse set of resource baits. Thirty 50 ml plastic tubes, holding one of six different nutrients in solution (H_2O, NaCl, glutamine, CHO (sucrose), CHO + glutamine, olive oil), were placed on the ground at times of peak ant activity and recollected with foraging ants after 2 h. All specimens were first identified to genus and then sorted into different morphospecies. For details, see Peters et al.[3].

Hymenoptera and hoverflies [with the exception of ants; data owners: A.C., W.J.K., R.S.P., C.D.E., M.K.P., I.S.D.]: Data on species richness of bees, other aculeate Hymenoptera (with exception of ants), parasitoid wasps and hoverflies were collected using pan traps[4,5]. A total of eight pan trap clusters, each consisting of one UV-bright blue, one yellow and one white pan were installed along two 50 m transects on each plot with a minimal distance of 15 m between clusters. We sampled pollinators in different vegetation heights, i.e. ~35 cm (herbal layer) and ~120 cm (shrub layer) above the ground. At study sites in forests we installed a subset of traps in the lower canopy (up to ~25 m). Pan traps were filled with water and a drop of liquid soap to break the water’s surface tension, and were recollected after 48 hours. Three sampling rounds were conducted summing up to a total of 24 pant trap clusters per plot. Due to the large number of specimens, for parasitoid wasps and other aculeate Hymenoptera only the specimens of two

sampling rounds were analyzed. Species were sorted to morphospecies level and, wherever possible, identified to species level. The group of parasitoid wasps included all apocritan Hymenoptera except Aculeata, and except Ichneumonidae, Eulophidae and Mymaridae. Specimens of the latter three groups were excluded as these were difficult to preserve and hardly identifiable on to morphospecies without specialized taxonomic expertise.

Moths [Heterocera; data owners: C.B., M.H.-B., M.T.]: Moths were caught using a custom-built automatic light trap with a superactinic light tube (6 watt, FRITZ WEBER Entomologiebedarf, Stuttgart, Germany). Wherever possible the trap was set up in the center of the study sites. On each study site the light trap was operated over four periods of 20 min (80 min in total), between 7 pm and 10 pm, starting 30 min after sunset. In all habitat types with at least occasional trees or shrubs, the trap was installed on an obstacle-free branch at a height of 1.5-2 m above the ground. In the treeless alpine zone the light trap was placed 0.3 m above ground. All sampled moths were dried and classified to morphospecies.

Dung beetles [subfamilies Scarabaeinae and Aphodiinae and genus *Trox* of the family Trogidae; data owners: F.G., I.S.D., M.K.P]: Dung beetles were collected with baited pitfall traps. On each study site one pitfall trap (upper diameter 33cm, lower diameter 24cm, height 15cm) was placed and equipped with 1.5 L of water and a drop of liquid soap to break water surface tension. Above traps 700 g of fresh cow dung was placed on a mesh. Cow dung was frozen for at least 24 hours prior to the experiment to make sure any dung beetles already in the dung were killed. Traps were left open for 72 h and after this time all captured specimens were sieved and stored in whirlpacks filled with 70% ethanol. In the laboratory dung beetles were sorted to families, then to morphospecies or species level.

Orthoptera [grasshoppers, locusts and bushcrickets; data owner: C.H.]: Orthoptera assemblages were recorded on all study sites by repeatedly walking for 1.5 h on parallel tracks (distance between transects ca. 1-1.5 m) and recording all sighted species. In forested study sites, trees and bushes in the understory vegetation were shaken for approximately 1.5 h. Insects falling from the vegetation were gathered on white canvas laid on the forest floor. Species which could not be identified during visits were collected and later identified. Study sites were also visited at night where Ensifera were registered acoustically. Additionally, two rounds of sweep net sampling were conducted on study sites to collect small species which may have remained undetected during transect walks. One round was conducted during the cool dry season (July to October) and one during the warm dry season (December to March). During each sweep netting round, 100 sweeps with a 30-cm diameter sweep were taken and all collected specimens were identified in the laboratory. Species accumulation curves for Caelifera and Ensifera on Mt. Kilimanjaro were published in Hemp[6,7] showing that more than 90% of the grasshopper, locust and bushcricket fauna for Mt. Kilimanjaro have been registered.

Ground-dwelling beetles [Coleoptera; data owners: J.R., R.B.]: Assemblages of ground-dwelling beetles were sampled with pitfall traps[8]. Ten pitfall traps were evenly spaced along two 50 m transects, with a distance of 10 m between individual traps and 20 m between transects. Pitfall traps were filled with 100-200 ml solution of equal parts of ethylenglycol and water with a drop of liquid soap to break the surface tension. The traps were placed on the sampling sites in June 2012 and collected after seven days. As the number of individuals collected in ten traps was very high and all individuals could not be analyzed in time, for the present analysis, we processed only three traps from each study site. Ground-dwelling beetles were sorted to morphospecies level, and where possible, to species.

True bugs [Heteroptera, data owner: M.K.P., J.T., J.D.]: True bugs were collected in two rounds of sweep net samplings. One round was conducted during the cool dry season (July to October) and one during the warm dry season (December to March). During each sweep netting round, 100 sweeps with a 30-cm diameter sweep were taken along two 50 m transects. All collected specimens were identified in the laboratory to families, then to species or morphospecies. Only data of adult specimens were used.

Collembola [springtails; data owners: J.R., R.B.]: For springtails we used the same sampling procedures as for the beetles (see above).

Ground-dwelling spiders [Araneae: data owner: M.H., J.R., R.B.]: Ground-dwelling spiders were collected from the same samples as the ground-dwelling beetles and Collembola. All adult and subadult spiders (74% of all spider individuals) were sorted to families and morphospecies.

Terrestrial gastropods [snails and slugs; data owners: C.N., R.B.]: To assess the species richness of terrestrial gastropods, a combination of two methods were applied at each sampling site[9–11]. First, we conducted four rounds of fixed-time surveys of 30 min in different seasons in which we intensively searched study sites for both living gastropods and empty shells. During these surveys we intensively searched all potential microhabitats of gastropods including the ground, the leaf litter, fallen tree trunks, under and on rocks and under bark. Second, we collected a total of 1L of leaf litter from different spots on each study site in order to collect gastropods of small size which may have remained undetected during fixed-time surveys. The litter was air-dried and sieved using a combination of stacked sieves of different mesh sizes (mesh size of top sieve = 2 mm and bottom 0.5 mm) and carefully inspected for shells using a stereomicroscope. Gastropods identification was based on external morphology. The use of the two methods allowed the

detection of both large-sized taxa that often occur at low density and micro-species that are cryptic and litter-dwelling.

Millipedes [Diplopoda: data owner: S.B.F., J.R., R.B.]: Millipedes were collected by a combination of pitfall trapping and repeated fixed-time (2 hours) intensive searches by hand. Pitfall trapping was done with five rounds of pitfall traps, most traps being placed in the small wet season around November or the months after the big wet season (June-September). Hand collecting was carried out in November-December and again in February - April, with dryer areas, such as savannah, being sampled when it was moist and green. Study sites were searched thoroughly by hand for two hours by searching places millipedes could conceivably be found, such as under rocks, dead wood or leaf litter. The collected millipedes were stored in 70% ethanol and identified in the laboratory. As male gonopods are crucial for determining the species, only data on adult male individuals was used.

Amphibians [Amphibia; data owners: G.Z., I.S.D., M.-O.R.]: Data on anurans were not collected on the same study sites like the other taxa but at 18 nearby study sites with lentic or lotic water sources which covered an elevational gradient from 905 m to 3548 m asl along the southern slopes of the mountain. Further surveys covered areas up to 4000 m asl. Above 3500 m, we did not find any amphibians. During diurnal and nocturnal random walks, we used a combination of visual and acoustic encounter surveys to search for frogs in all microhabitats[12]. All visits were randomly distributed during the sampling periods, and all sites were visited at least three times. For details, see Zancolli et al.[13].

Birds [Aves; data owners: S.W.F., K.B.-G.]: We used audiovisual point counts on eight subplots per study site to record birds[14]. We established circles with a 20-m radius in densely vegetated habitats (savannah and all forest habitats) and 35.5 m × 35.5 m squares at alpine *Helichrysum*

sites, covering the same sampling area in all habitat types. Point counts started 15 min before sunrise and were completed before 9 am. All birds heard or seen in one subplot were counted for 10 min and identified[15]. Birds were counted in all strata, including the ground, the lower vegetation, the tree canopy and above the tree canopy. Birds were surveyed twice per study site, once during the cool dry season (July to October) and once during the warm dry season (December to March). All 480 point counts (30 study sites × 8 subplots × 2 seasons) were conducted by the same observer to reduce inter-observer variability.

Aerial insectivorous bats [Chiroptera; data owners: M.H.-B., M.T.]: Species richness of aerial insectivorous bats was assessed by acoustic monitoring using a standardized point stop method at the four corners of the study sites[16]. Every corner was visited for five minutes and echolocation calls of all passing bats were recorded manually using a real time ultrasound recorder (Pettersson D1000x). All four corners of one site were visited four times between local sunset and 11:30 pm, resulting in 80 min recording per study site per night. Echolocation calls were classified into sonotypes based on call characteristics (start and end frequency, the frequency with the highest amplitude, call duration and intervals between calls). The number of distinct sonotypes recorded per study site was used as a measure of bat species richness.

Supplementary References

1. Chao, A. & Jost, L. Coverage-based rarefaction and extrapolation: standardizing samples by completeness rather than size. *Ecology*, **93**, 2533-2547 (2012).
2. Braun-Blanquet, J. *Pflanzensoziologie: Grundzüge der Vegetationskunde*. (Springer-Verlag, 1964).

3. Peters, M. K., Mayr, A., Röder, J., Sanders, N. J. & Steffan-Dewenter, I. Variation in nutrient use in ant assemblages along an extensive elevational gradient on Mt Kilimanjaro. *J. Biogeogr.* **41,** 2245–2255 (2014).

4. Classen, A. *et al.* Temperature versus resource constraints: which factors determine bee diversity on Mount Kilimanjaro, Tanzania?: Bee species richness on Mt Kilimanjaro. *Glob. Ecol. Biogeogr.* **24,** 642–652 (2015).

5. Westphal, C. *et al.* Measuring bee diversity in different European habitats and biogeographical regions. *Ecol. Monogr.* **78,** 653–671 (2008).

6. Hemp, C. Annotated list of Caelifera (Orthoptera) of Mt. Kilimanjaro, Tanzania. *J. Orthoptera Res.* **18,** 183–214 (2009).

7. Hemp, C. Annotated list of Ensifera (Orthoptera) and further records on Caelifera (Orthoptera) of Mt Kilimanjaro, Tanzania. *Zootaxa* **3613,** (2013).

8. Röder, J., Detsch, F., Otte, I., Appelhans, T., Nauss, T., Peters, M.K. & Brandl, R. (*in press*) Heterogeneous patterns of abundance of epigeic arthropod taxa along a major elevation gradient. *Biotropica*. DOI: 10.1111/btp.12403

9. Emberton, K. C., Pearce, T. A. & Randalana, R. Quantitatively sampling land-snail species richness in Madagascan rainforests. *Malacologia* **38,** 203–212 (1996).

10. Emberton, K. C., Pearce, T. A., Kasigwa, P. F., Tattersfield, P. & Habibu, Z. High diversity and regional endemism in land snails of eastern Tanzania. *Biodivers. Conserv.* **6,** 1123–1136 (1997).

11. Tattersfield, P. Local patterns of land snail diversity in a Kenyan rainforest. *Malacologia* **38,** 161–180 (1996).

12. Rödel, M.-O. & Ernst, R. Measuring and monitoring amphibian diversity in tropical forests. I. An evaluation of methods with recommendations for standardization. *Ecotropica* **10,** 1–14 (2004).

13. Zancolli, G., Steffan-Dewenter, I. & Rödel, M.-O. Amphibian diversity on the roof of Africa: unveiling the effects of habitat degradation, altitude and biogeography. *Divers. Distrib.* **20,** 297–308 (2014).

14. Ferger, S. W., Schleuning, M., Hemp, A., Howell, K. M. & Böhning-Gaese, K. Food resources and vegetation structure mediate climatic effects on species richness of birds: Climate and bird species richness. *Glob. Ecol. Biogeogr.* **23,** 541–549 (2014).

15. Zimmerman, D. A., Turner, D. A. & Pearson, D. J. *Birds of Kenya and Northern Tanzania*. (Princeton University Press, 1999).

16. Helbig-Bonitz, M., Ferger, S. W., Böhning-Gaese, K., Tschapka, M., Howell, K., & Kalko, E. K. (2015). Bats are not birds - different responses to human land-use on a tropical mountain. *Biotropica*, ***47***, 497-508.
